# Microencapsulated Botanicals and Organic Acids Improve Immune Status and Growth in Gilthead Seabream (*Sparus aurata* L.)

**DOI:** 10.1155/anu/4213038

**Published:** 2026-01-07

**Authors:** Fabrizio Caruso, Andrea Toschi, José María García-Beltrán, Alberto Cuesta, Costanza Bonnici, Andrea Piva, María Ángeles Esteban, Ester Grilli

**Affiliations:** ^1^ Innovation Department, Vetagro S.p.A., Reggio Emilia, 42124, Italy; ^2^ Immunobiology for Aquaculture Group, Department of Cell Biology and Histology, Faculty of Biology, University of Murcia, Murcia, 30100, Spain, um.es; ^3^ DIMEVET, University of Bologna, Ozzano Emilia, Bologna, 40064, Italy, unibo.it; ^4^ Innovation Department, Vetagro Inc., 936 SW 1st Avenue Suite 878, Miami, 33130, Florida, USA

**Keywords:** feed additives, fish nutrition, health status, phytogenics, thymol, vanillin

## Abstract

For their ability to enhance performance, immune response, and robustness to environmental stressors in both fish and crustaceans, phytogenic compounds are receiving increasing attention from the aquaculture industry as alternatives to traditional feed additives. Numerous studies have investigated the use of a specific combination of organic acids and botanicals (OA + B) in terrestrial animals, but their potential role in aquaculture remains unexplored. The objective of this study is to assess the feasibility of a blend of OA + B (microencapsulated in a lipid matrix; AviPlusAqua – Vetagro S.p.A.) to enhance the health of gilthead seabream. To better assess the potential of the selected blend, both in vitro and in vivo experiments were conducted. Head‐kidney leukocytes (HKLs) were incubated with varying doses of OA + B, then viability and cellular immune parameters were evaluated after 30 min, 2 h, and 4 h. For the in vivo assay, 120 gilthead seabreams (body weight [BW]: 48.00 ± 5.00 g) received a diet supplemented with 0 (control [CTR]), 250, or 500 ppm of OA + B; then growth performance, humoral and cellular immunity, and gene expression of immune‐related genes were evaluated after 15, 30, and 60 days. In vitro, data from gene expression, phagocytosis, and respiratory burst assays demonstrated that OA + B positively stimulate HKLs activity. In vivo results showed increased growth performance (+19% in overall BW; +0.31 specific growth rate [SGR]) from 30 days of supplementation onward, along with improved humoral and cellular immunity. Gene expression analysis of intestinal samples revealed a positive modulation of genes related to intestinal oxidative stress response and a balanced pro‐/anti‐inflammatory cytokine profile at both tested dosages. The results highlight that dietary OA + B supplementation modulates the immune response under homeostatic conditions, as evidenced by modulated expression of immune‐related genes and enhanced phagocytic and respiratory burst activities.

## 1. Introduction

In the last years, aquaculture has established its role as a pivotal and vital sector in global food production and provision, developing worldwide along with the increasing demand for protein [1]. Among the many aquatic species cultivated in the Mediterranean and Black Sea region, the gilthead seabream (*Sparus aurata* L.) holds a prominent position due to its production volumes, adaptability to different environmental conditions, and market appreciation [2, 3]. Since the early 2000s, gilthead seabream production has expanded significantly, making this finfish one of the main players in Mediterranean aquaculture in both volume and economic value (281,914.00 tons, 34.1% of total aquaculture production; [4]). The intensification of farming practices, however, has posed several challenges since the early development of this industry [5]. Environmental and human stressors, bacterial diseases, and parasitic outbreaks have been widely reported in the literature for this species, highlighting negative impacts on fish health and overall performance, with consequent economic losses [6, 7].

Therefore, it has become essential to explore new strategies to increase resilience against daily challenges while maintaining the sustainability of aquaculture activities [8, 9]. One possible approach to consider is the use of functional feeds, which involve enriching diets with functional ingredients capable of modulating various aspects of fish metabolism [10, 11].

Among the various alternatives within the feed additives category, botanical compounds have attracted increasing interest in the industry for their ability to enhance performance, immune response, and robustness to environmental stressors in both fish and crustaceans [12]. In particular, botanicals represent attractive options due to their antioxidant, antimicrobial, and immunomodulatory effects [13]. In aquatic species, plant extracts have been successfully incorporated into feeds to enhance growth performance, improve gut health, and boost immune responses [14] due to their capacity to mitigate oxidative stress and reduce the prevalence of bacterial infections, while supporting intestinal microbiota balance [15, 16]. Another promising class of molecules is organic acids (OAs), which have a well‐known ability to improve growth performance and feed utilization as a result of their capacity to influence pH, nutrient absorption, gut microflora, and ultimately decrease the prevalence of disease‐causing pathogens [17–19].

The use of a precise combination of OA and botanicals (OA + B) has been widely explored both in Caco‐2 cell in vitro models and extensively in vivo in terrestrial livestock, with special reference to swine and poultry production. In vitro studies using Caco‐2 epithelial cell models have demonstrated that this blend enhances intestinal barrier function and tight junction protein expression [20–22]. In terrestrial livestock, dietary supplementation of these compounds promoted growth under challenging conditions, beneficially modulated the microbiome, and often improved feed conversion efficiency [20, 23, 24]. To date, the role of this blend remains a relatively unexplored area in aquaculture. The available literature on aquatic species reports beneficial modulation of the microbiome with proliferation of beneficial lactic acid bacteria, the upregulation of the anti‐inflammatory cytokines interleukin‐10 (*il-10*) and transforming growth factor beta (*tgf-β*) gene expression, and the potential to mitigate stress, although with not comparable results, in two different studies with rainbow trout (*Oncorhyncus mykiss*) and European seabass (*Dicentrarchus labrax*) [25, 26]. Finally, in whiteleg shrimp (*Litopenaeus vannamei*), OA + B not only beneficially modulated the microbiome but also enhanced robustness with increased relative expression of immune‐related genes and overall survival in a bacterial challenge [27].

To the best of our knowledge, there are no prior studies on gilthead seabream concerning the application of an OA + B blend. Consequently, this study aimed to assess the feasibility of utilizing this blend to enhance the health of gilthead seabream. Specifically, both in vitro and in vivo analyses were conducted to investigate the effects of the active ingredients directly on head‐kidney leukocytes (HKLs). Additionally, the study examined the effects of the formula, when incorporated into dietary treatments in its microencapsulated form, on growth performance, as well as on humoral and cellular immunity in gilthead seabream.

## 2. Materials and Methods

### 2.1. Ethical Statement

All animal procedures follow guidelines established by the Ethical Committee for Animal Experimentation of the University of Murcia and were approved by this committee (Permit Number A13160416). The experiments were conducted in accordance with the Guiding Principles for Biomedical Research Involving Animals (European directive 2010/63/EU) on the protection of animals used for scientific purposes.

### 2.2. In Vitro Screening on HKLs

Ten specimens of 50 ± 5 g mean body weight (BW) of gilthead seabream, obtained from a local farm (Murcia, Spain), were kept in two marine aquaria at the Marine Fish Facilities at the University of Murcia (250 L, flow rate 900 L/h) at 28‰ salinity, 20°C, under a 12 h light/12 h dark photoperiod, and maintained in quarantine for 4 weeks. Fish were fed twice daily with a commercial pellet diet that met or exceeded the established nutrient requirements [28] at a rate of 2% of BW per day as recommended by the manufacturer and were acclimated for 15 days prior to the in vitro experiments. The fish were euthanized after 24 h starving by using an overdose of benzocaine (4% in acetone, Sandoz) [29].

HKLs were isolated as described by Esteban et al. [30]. Briefly, HK tissue from euthanized fish was excised, cut into small fragments, and placed in 8 mL of sRPMI (Roswell Park Memorial Institute medium), which consists of: RPMI‐1640 culture medium (Gibco) supplemented with 3% fetal calf serum (FCS, Gibco), 100 I.U./mL penicillin (Flow), 100 mg/mL streptomycin (Flow), and 0.35% NaCl to match gilthead seabream plasma osmolarity. Tissue fragments were forced through a nylon mesh (mesh size 100 μm), and resulting cells were washed twice (400 g x 10 min, 22°C), and counted using an automatic counting chamber (BioRad) to adjust the concentration to 2 × 10^7^ cells/mL in sRPMI. Cell viability was determined by the trypan blue exclusion test.

The possible effect of different concentrations of a blend of OA + B on HKLs activities was assessed. The HKLs were incubated with increasing dosages (50, 100, 250, and 500 ppm) of a mixture of citric and sorbic acids (25% and 16.7%, respectively), thymol (1.7%), and vanillin (1%). The active ingredients and doses were selected to meet the composition of the product used in the experimental diets of the in vivo trial and in a previous study [21]. Cells were incubated in the absence (control [CTR]) or presence of OA + B at 20°C in an incubator with 5% CO_2_ and 85% humidity for four different time‐points (0 min, 30 min, 2 h, and 4 h). After incubation, HKLs viability was evaluated to determine the possible cytotoxic effect of the blend [31]. Briefly, 40 µL of propidium iodide (PI) (400 mg/mL, Sigma–Aldrich, St. Louis, Missouri, USA) was added to each aliquot of 100 µL of HK leukocytes incubated for 30 min, 2 h, or 4 h without CTR or with the OA + B blend. For each time‐point, the tubes were gently mixed and subsequently analyzed using a FACScan (Becton Dickinson, Madrid, Spain) flow cytometer with an argon‐ion laser adjusted to 488 nm. Analyses were performed on 5000 cells, which were acquired at a rate of 300 cells/s. Data were collected in the form of two‐parameter side scatter (SSC) (granularity), and forward scatter (FSC) (size), and green fluorescence (FL1) and red fluorescence (FL2) dot plots or histograms were generated using a computerized system. Dead cells were expressed as the percentage of cells with PI (red‐PI fluorescent cells). Subsequently, noncytotoxic concentrations of OA + B were used for phagocytic parameters, respiratory burst, and gene expression analysis of immune‐related genes as described in the following paragraphs.

### 2.3. In Vivo Study

#### 2.3.1. Experimental Diets

A commercial diet (VITA 2, Veronesi, A.I.A SpA, Verona, Italy, pellet diameter 2.3–2.6 mm) for gilthead seabream formulated including practical ingredients for marine mediterranean species (fishmeal, wheat gluten, soy protein concentrate, fish oil, wheat, soybean oil, and soybean meal) and containing: 49.8% protein; 21.6% lipid; 7.3% ash; 5.6% moisture and with an energy content of 5152.6 cal/g was selected as CTR. Analytical composition is provided in Table S1. Then the two treated diets (D250 and D500) were obtained by top‐coating the CTR diet with 250 and 500 ppm of the microencapsulated blend, respectively. The blend in study is a mix of OA + B composed of 25% citric acid, 16.7% sorbic acid, 1.7% thymol, and 1% vanillin, encapsulated in a matrix of hydrogenated fats (AviPlusAqua – Vetagro SpA, Reggio Emilia, Italy; US patent # 7,258,880; EU patent # 1‐391‐155B1; CA patent # 2,433,484).

#### 2.3.2. Experimental Design

One hundred and twenty gilthead seabream specimens with an average BW of 48.00 ± 5.00 g were obtained from a private local farm (Spain), acclimatized, and maintained as previously described. Fish were randomly allocated to six aquaria, with each aquarium containing 20 fish, and two replicates were established for each treatment. Each aquarium (250 L, flow rate 900 L/h) was then randomly assigned to one of the three diets under investigation. The fish were fed to satiation twice daily, at 09:00 and 17:00 h, over a period of 60 days, and the feed consumption in each tank was recorded.

#### 2.3.3. Sampling Activities

For the evaluation of immune parameters, five fish from each aquarium were sampled after 15 and 30 days of feeding. Immune indicators were assessed at days 15 and 30 to capture early and intermediate responses to dietary changes, whereas at day 60, only growth performance was evaluated, as immune responses were expected to stabilize. The HK was dissected, sectioned into small fragments, and transferred to 8 mL of sRPMI for leukocyte isolation. Once isolated, HKLs were assessed for phagocytic activity and respiratory burst. The proximal intestine was collected and preserved in TRIzol Reagent (Invitrogen) at −80°C for subsequent gene expression analysis. For the assessment of humoral immune responses, blood samples were collected from the caudal blood vessels with an insulin syringe and were allowed to clot at 4°C for 4 h. The serum was collected after centrifugation (10,000 × *g*, 10 min, 4°C) and stored at −80°C.

#### 2.3.4. Additive Inclusion and Feed Validation

To determine the correct inclusion of the blend in feed, the method was adapted from a study by Ozdemir et al. [32]. For sorbic acid determination, 50.00 ± 0.01 g of feed were sampled and added to 450 mL of 32 mM KOH. The mixture was then stirred at 70°C for 30 min, cooled to room temperature (RT), and centrifuged. Then, 70 mL of distilled water was added to 10 mL of supernatant, adjusting the pH to 4.5 using 3N HCl. The solution was then filtered through a 0.45 µm syringe filter (Interchim, Montluçon, France) for chromatographic analysis. For thymol determination, 5.00 ± 0.01 g of feed were sampled and added with 5 mL of diethyl ether (Carlo Erba, Milan, Italy) and 5 mL of absolute ethanol. The mixture was stirred for 2 min at RT, then immersed in a water bath at 65°C for 5 min. The tube was centrifuged, then the supernatant was collected and filtered through a 0.45 µm syringe filter for chromatographic analysis.

The HPLC analysis was carried out on a Jasco LC‐4000 series HPLC system (Easton, Maryland, USA). UV detection was performed at 253 nm for sorbic acid and 270 nm for thymol, with a deuterium (D2) lamp. Analyses were run using an X‐Terra RP‐18 column (Water, Milford, Massachusetts, USA). The mobile phase was 1% H_3_PO_4_ (solvent A) and acetonitrile (solvent B), separation occurred with a linear gradient in 40 min, with a 1 mL/min flow rate. Standards of sorbic acid and thymol were used of the identification and quantification (Sigma–Aldrich, St. Louis, Missouri, USA).

#### 2.3.5. Growth Performance

BW of each fish was measured prior to the initiation of the trial and subsequently on days 15, 30, and finally on day 60, which marked the conclusion of the study. Growth was monitored by obtaining the initial weight (Wi), final weight (Wf), weight gain (WG), percentage of WG (%WG), and specific growth rate (SGR). Fish were fed ad libitum, and the feed conversion ratio (FCR) was monitored by obtaining the amount of feed ingested (FI) and correlating it to the WG. Parameters were calculated for each group and timepoint according to Silva‐Carrillo et al. [33]:

%WG=Wf−Wi/Wi×100,

SGR=Ln final weight− Ln initial weight number of days−1×100,

FCR= FI/WG.

#### 2.3.6. Humoral Immunity

Alternative complement pathway activity was assayed in serum using sheep red blood cells (SRBC, Biomedics) as the target, as previously described by [34]. Briefly, a 6% SRBC suspension was mixed with serially diluted serum to give final serum concentrations ranging from 10% to 0.078%. The dilutions were incubated for 90 min at 22°C, after which the relative hemoglobin content was quantified by measuring optical density at 550 nm (BMG Labtech GmbH, Germany). The serum volume required to produce 50% hemolysis (ACH_50_) was determined, and values were expressed as ACH_50_ units/mL for each fish.

Total serum immunoglobulin M (IgM) levels were analyzed using an enzyme‐linked immunosorbent assay (ELISA) adapted from a study by Cuesta et al. [35]. Diluted serum samples (1:500, 20 µL/well, in triplicate) were coated in carbonate‐bicarbonate buffer (35 mM NaHCO_3_ and 15 mM Na_2_CO_3_, pH 9.6) overnight at 4°C. After washing with PBS (phosphate‐buffered saline) + 0.05% Tween 20 (PBT, Sigma–Aldrich, St. Louis, Missouri, USA), plates were blocked for 2 h at RT with blocking buffer containing 3% bovine serum albumin (BSA, Sigma–Aldrich, St. Louis, Missouri, USA). Then plates were then incubated for 1 h with 100 μL of mouse antigilthead seabream IgM monoclonal antibody (Aquatic Diagnostics Ltd, United Kingdom) (1/100 in blocking buffer), and then with secondary antibody anti‐mouse IgG‐HRP (1/1000 in blocking buffer, Sigma–Aldrich, St. Louis, Missouri, USA). Signal development was achieved using 100 μL of a 0.42 mM TMB solution for 10 min, and the plates were read at 450 nm. The absorbance of negative CTRs (without serum) was subtracted from each sample value.

### 2.4. Cellular Immunity

The phagocytic activity, respiratory burst, and gene expression analysis of immune markers were performed to evaluate the effect of the blend on cellular immunity. For the in vitro assay, cellular immunity was evaluated on HKLs incubated for 30 min, 2 h, and 4 h with 0, 50, 100, 250, or 500 ppm of OA + B. For the in vivo analysis, cellular immunity was evaluated on HKLs isolated from fish fed CTR, D250, or D500 for 15 and 30 days. In this case, the gene expression analysis for immune markers was performed on proximal intestinal samples.

The phagocytic activity of gilthead seabream HKLs was analyzed by flow cytometry following the previous design [30]. Heat‐killed (30 min, 60°C) lyophilized S*accharomyces cerevisiae* (strain S288C) was washed twice, counted, adjusted to 10^8^ cells/mL in sRPMI‐1640, and labeled with fluorescein isothiocyanate (FITC, Sigma–Aldrich, St. Louis, Missouri, USA) [34]. After labeling, free FITC was removed by washing twice in PBS, and the FITC‐labeled yeast cells were resuspended in sRPMI‐1640 for flow cytometric analysis. For phagocytosis, 60 µL of labeled‐yeast cells and 100 µL of HKLs were mixed, centrifuged (400 *g* x 5 min, 22°C), resuspended, and incubated at 22°C for 30 min under dark conditions. To stop phagocytosis, samples were placed on ice, and 400 µL ice‐cold PBS was added, extracellular fluorescence (free yeast cells and yeast cells adhered to phagocytes but not ingested) was quenched by adding 50 µL of ice‐cold trypan blue (0.5% in PBS). Standard samples of FITC‐labeled *S. cerevisiae* or HKLs were included in each phagocytosis assay. All samples were analyzed using a flow cytometer (Becton Dickinson) with an argon‐ion laser adjusted to 488 nm. Data were acquired as two‐parameter SSC (granularity) and FSC (size), and green fluorescence (FL1) dot plots or histograms were generated using a computerized system. Phagocytic ability was defined as the percentage of cells with one or more ingested yeast cells (green‐FITC fluorescent cells), while phagocytic capacity corresponded to mean fluorescence intensity. Data were processed using the Lysis Software Package (Becton Dickinson, New Jersey, USA).

The respiratory burst activity of HKLs was determined by chemiluminescence, according to Bayne and Levy [36]. HKLs (100 µL) were incubated with 100 µL of HBSS (Hank’s balanced salt solution, Gibco) containing 1 mg/mL phorbol myristate acetate (PMA, Sigma–Aldrich, St. Louis, Missouri, USA) and 10 mM luminol. Plates were shaken and then immediately read using a chemiluminometer (BMG, FluoStar Galaxy, BMG Labtech GmbH, Germany) for 30 cycles of 2 min each. The kinetics of the reactions were recorded, and the maximum slope of each curve was calculated.

### 2.5. Gene Expression Analysis

Total RNA was extracted from HKLs or the proximal intestine using TRIzol Reagent. The concentration and purity were evaluated using a Nanodrop (Thermo Fisher Scientific, Massachusetts, USA) before treating with DNase I (Promega, Promega Biotech Ibérica, Spain) to remove contamination from genomic DNA. Complementary DNA (cDNA) was synthesized. The expression of genes relevant to inflammatory response and antioxidant activity was determined by real‐time PCR (QuantStudio 5, Applied Biosystems) using SYBR Green qPCR Master Mix (Applied Biosystems). The target genes included: nuclear factor erythroid‐2 (*nrf2*), a master regulator of antioxidant defense; superoxide dismutase (*sod*) and glutathione reductase (*gr*), involved in reactive oxygen species (ROS) detoxification; interleukin‐8 (*il-8*), a pro‐inflammatory chemokine; and *il-10*, an anti‐inflammatory cytokine. The primers used are shown in Table S2. The relative expression of all genes was calculated by the 2^− ΔΔCT^ method [37], using *gilthead seabream* 18S ribosomal RNA (*18S*) and elongation factor 1‐alpha (*ef1α*) as housekeeping genes.

### 2.6. Statistical Analysis

All assays were performed in triplicate. Data are presented as mean ± standard error mean (*SEM*), except growth performance data, which are reported as mean ± standard deviation (SD). One‐way ANOVA was used to test differences between all treatments, and a post hoc test (Tukey’s) for comparison of means was used to test the differences between two specific treatments when necessary. Statistical analyses were carried out using GraphPad Prism v.10.4.1 (GraphPad Software, Inc., Boston, Massachusetts, USA). The Shapiro–Wilk and Kolmogorov–Smirnov tests were performed for normality and homogeneity checks, respectively. Differences were considered significant when *p* ≤ 0.05, and trends were identified when 0.05 < *p* ≤ 0.1.

## 3. Results

### 3.1. In Vitro Screening

#### 3.1.1. HKL Viability

HKL viability was tested after 30 min, 2 h, and 4 h of incubation with the blend at different concentrations ranging from 0 ppm (CTR) to 500 ppm of OA + B. The results indicated that no significant effect was observed on HKL viability following incubation with the blend (Figure S1). As no cytotoxic effect was reported, all concentrations were also used for cellular immunity evaluation.

#### 3.1.2. HKLs Phagocytosis and Respiratory Burst

After incubation with OA + B, HKLs showed a significantly increased phagocytic ability at 500 ppm after 30 min (Figure 1a), no differences at 2 h (Figure 1b), while at the end of the incubation period, only 100 ppm showed a significant increment (Figure 1c), compared to the CTR. Additionally, the phagocytic capacity, representing the number of particles per phagocytic cell, was reported as a percentage over the initial fluorescence intensity value (time 0). The phagocytic capacity of HKLs was significantly increased compared to the CTR during incubation with 50 ppm of OA + B at all time‐points (Figure 1). A significant increase in respiratory burst rate occurred after 30 min of incubation with OA + B at all tested doses, which remained constant throughout the entire treatment period (Figure 2).

Figure 1Phagocytic ability and capacity of head‐kidney (HK) leukocytes incubated for 30 min (a, d), 2 h (b, e), and 4 h (c, f) with incremental doses of the blend of OA + B. Data in the figures are means (*n* = 5) ± *SEM* represented by vertical bars. Means with different letters indicate statistical significance with *p*  < 0.05 (a, b).

**Figure:**
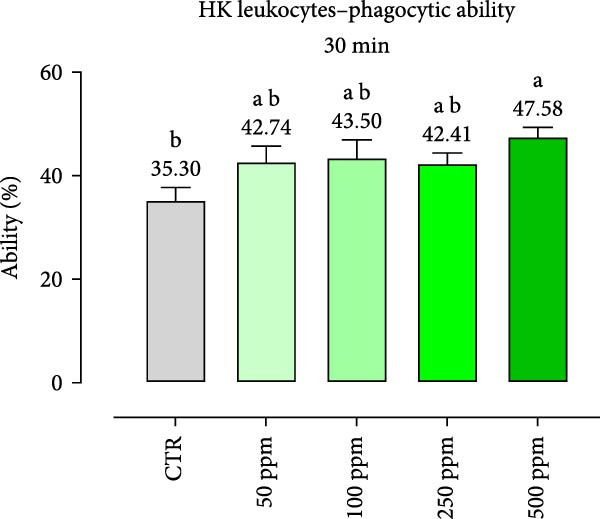
(a)

**Figure:**
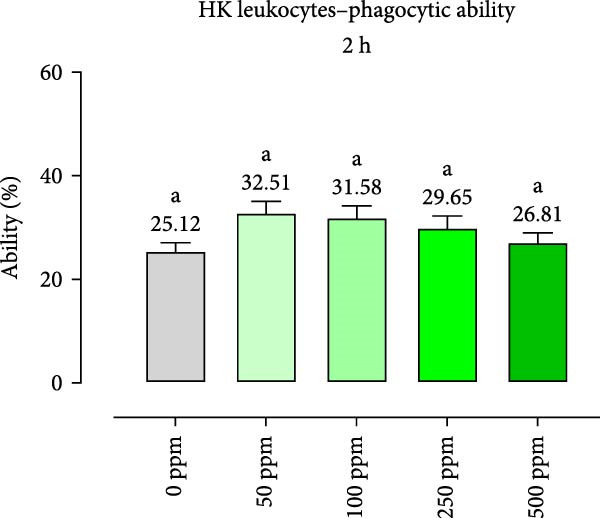
(b)

**Figure:**
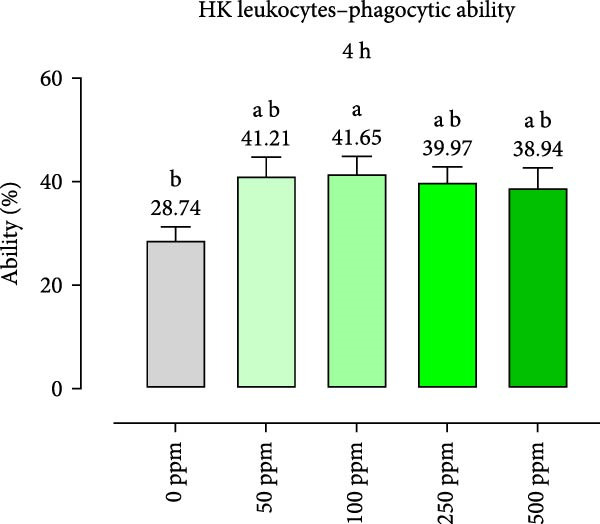
(c)

**Figure:**
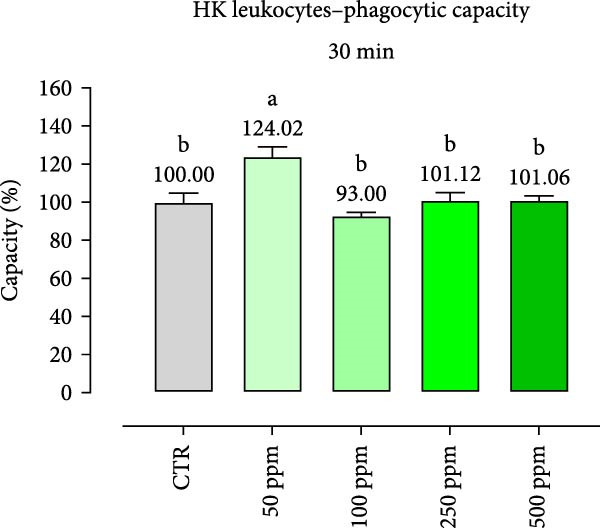
(d)

**Figure:**
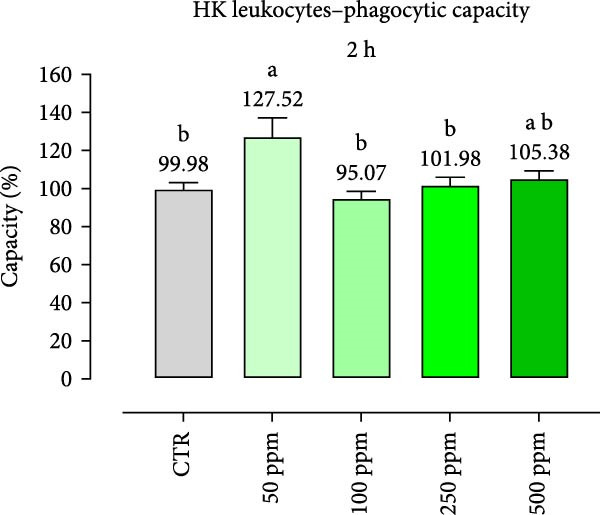
(e)

**Figure:**
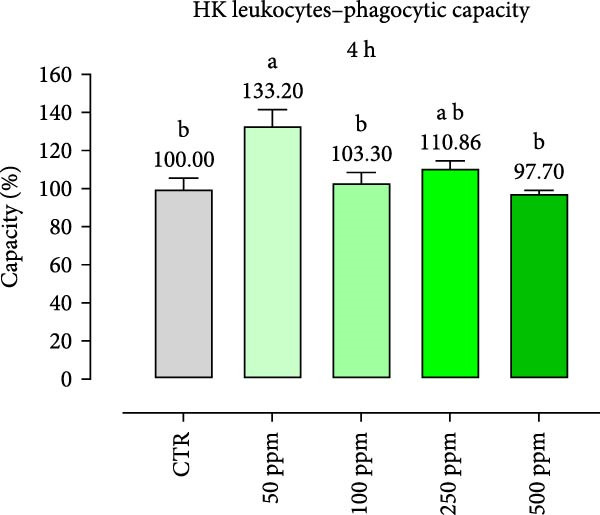
(f)

Figure 2Respiratory burst of head‐kidney (HK) leukocytes treated for 30 min (a), 2 h (b), and 4 h (c) with incremental doses of the blend of OA + B. Data in the figures are means (*n* = 5) ± *SEM* represented by vertical bars. Means with different letters indicate statistical significance with *p*  < 0.05 (a, b).

**Figure:**
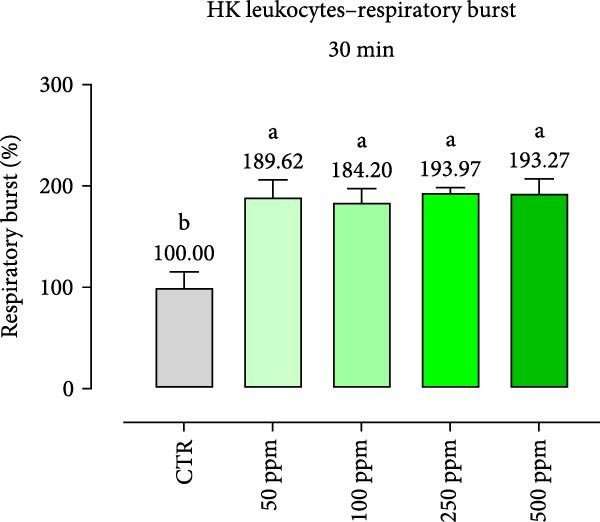
(a)

**Figure:**
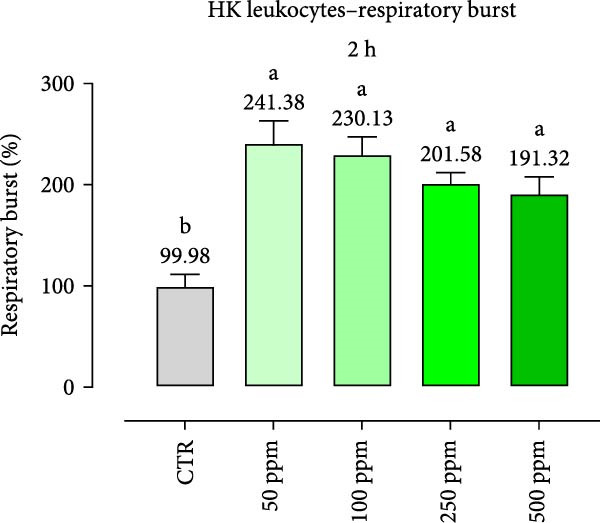
(b)

**Figure:**
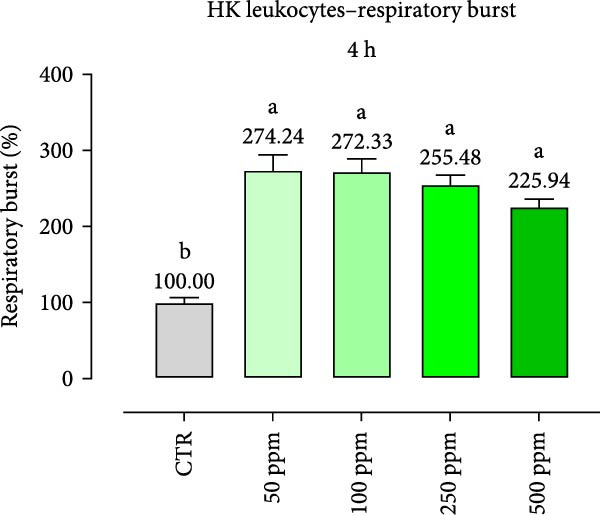
(c)

#### 3.1.3. HKLs Gene Expression of Immune Markers

Gene expression results of immune markers (*nrf2*, *sod*, *gr*, *il-7*, *il-8*, *il-10*) at two different timepoints (2 and 4 h) are presented in Figure 3. The results demonstrate dose‐ and time‐dependent effects of OA + B on HKLs. HKLs treated with at least 100 ppm of OA + B showed a general tendency to increase antioxidant markers (*nrf2*, *sod*, *gr*). Compared to the CTR at 2 h, *gr* and *nrf2* were upregulated at 250 ppm (fold changes of 2.10 and 1.77, respectively) while *sod* was enhanced at 500 ppm (fold change of 1.70). However, with prolonged exposure (4 h), gene expression of *gr* increased at all doses (fold change of 1.77 at 50 ppm of inclusion), *sod* was enhanced starting at 100 ppm (fold change 1.58), and *nrf2* from 250 ppm onward (fold change of 1.62). Regarding pro‐inflammatory cytokines, *il-7* and *il-8* were downregulated after 2 h starting from 50 ppm and 250 ppm onward (fold changes of 0.47 for both), respectively. At 4 h, *il-7* decreased from 250 ppm (fold change of 0.58), while *il-8* was not affected. Conversely, the anti‐inflammatory cytokine *il-10* was upregulated at all doses at 4 h (fold changes between 1.65 and 1.87). These data highlight the blend’s potential to modulate inflammatory tone with low doses and prolonged application.

Figure 3mRNA levels of antioxidant and inflammatory markers in head‐kidney leukocytes incubated 2 h (a) and 4 h (b) with incremental doses of the blend of OA + B. *nrf2* = nuclear factor erythroid 2; *sod* = superoxide dismutase; *gr* = glutathione reductase; *il* = interleukin. Data in the figures are means (*n* = 4) ± *SEM* represented by vertical bars. Means with different letters indicate statistical significance with *p*  < 0.05 (a, b).

**Figure:**
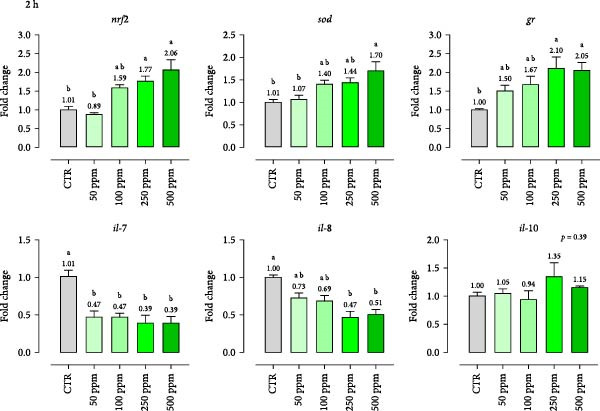
(a)

**Figure:**
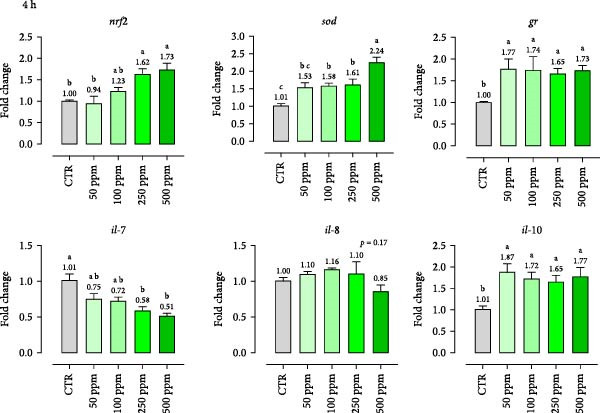
(b)

### 3.2. In Vivo Assay

For the in vivo study, the possible effect of dietary administration of the blend in its microencapsulated form was studied in gilthead seabream specimens with a specific focus on growth performance, conversion efficiency, and immune status. The feed was previously validated, and the results are provided in Table 1.

**Table 1:** Additive biomarkers recovery via extraction and HPLC analysis of aquafeed diets (*n* = 4).

Diets	Biomarker 1—thymol			Biomarker 2—sorbic acid		
	Recovery (%)	SD	CV	Analysis (%)	SD	CV
*CTR*	<LOD	—	—	<LOD	—	—
*D250*	97.52	5.41	3.57	103.01	6.71	0.43
*D500*	96.28	4.74	2.19	98.23	1.57	2.37

*Note:* CTR diet is detailed in Section 2.3.1 and in Table S1.

Abbreviations: CV, coefficient of variation; LOD, limit of detection; SD, standard deviation.

#### 3.2.1. Growth Performance

Growth performance results are presented in Table 2. Fish fed D500 at day 30 showed higher final BW than those fed the CTR diet or D250. However, at day 60, a similar difference relative to CTR was recorded for fish fed D250, with no statistical differences between the treated diets. SGR and WG% analysis highlighted strong differences relative to CTR for both treated diets at day 30; however, these differences were reduced at day 60, maintaining statistical differences solely with diet D500. All treated diets showed high intake and acceptability by the fish, with no significant differences in feed efficiency parameters among treatments. Notably, by day 60, fish fed the treated diets showed improved FCR compared to those fed the control diet.

**Table 2:** Growth performance parameters of gilthead seabream fed different diets with increasing levels of OA + B at three different timepoints.

Parameter	Diet	Time‐point									
		Day 15			Day 30			Day 60			
*BW (gr)*	CTR	51.70	±	9.62	60.00	±	10.67^b^	91.38	±	12.99^b^	
	D250	49.20	±	12.68	61.70	±	5.87^b^	104.5	±	15.81^ab^	
	D500	50.20	±	4.367	80.20	±	14.20^a^	109.0	±	7.76^a^	
	*p*‐Value	ns			<0.01			<0.05			
*WG (%)*	CTR	14.18	±	13.29	11.52	±	3.84^c^	88.83	±	25.12^b^	
	D250	13.14	±	18.66	37.59	±	15.44^b^	115.95	±	30.56^ab^	
	D500	8.84	±	8.02	55.13	±	8.87^a^	125.25	±	14.86^a^	
	*p*‐Value	ns			<0.001			<0.01			
*SGR*	CTR	0.85	±	0.73	1.02	±	0.55^c^	1.04	±	0.23^b^	
	D250	1.14	±	1.23	2.10	±	0.71^b^	1.27	±	0.23^ab^	
	D500	0.55	±	0.48	3.06	±	0.58^a^	1.35	±	0.11^a^	
	*p*‐Value	ns			<0.001			<0.05			
*FCR*	CTR	2.40	±	0.97	1.45	±	1.28	1.36	±	0.20	
	D250	1.39	±	2.19	2.18	±	0.34	1.24	±	0.29	
	D500	0.15	±	5.35	1.05	±	0.14	1.19	±	0.06	
	*p*‐Value	ns			ns			ns			

*Note:* Data represent mean ± SD (*n* = 10). One‐way ANOVA was used to test differences between all treatments at each timepoint, and Tukey’s post hoc test was applied when a significant interaction occurred. Different letters denote significant differences between experimental treatments (*p*  < 0.05).

Abbreviations: BW, body weight; FCR, feed conversion rate; SGR, specific growth rate; WG, weight gain.

#### 3.2.2. Humoral Immunity

Two humoral immune parameters, natural hemolytic complement activity and total IgM levels, were determined for each sampled fish fed the different diets at days 15 and 30 of this study and are presented in Figure 4. The complement activity in serum from fish fed the different diets for 15 days was consistently numerically lower than the values recorded for fish fed the same experimental diet for 30 days, but no differences were detected among treatments (Figure 4).

Figure 4Serum Immunoglobulin M (IgM) and natural hemolytic complement activity from fish fed for 15 days (a, c) and 30 days (b, d) with CTR, D250, or D500 diets. Data in the figures are means (*n* = 5) ± *SEM* represented by vertical bars. Means with different letters indicate statistical significance with *p*  < 0.05 (a, b). OD = optical density; ACH50 = serum alternative complement activity.

**Figure:**
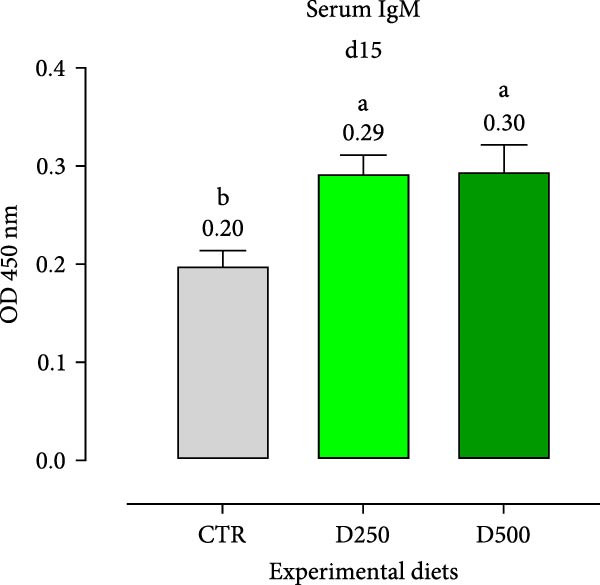
(a)

**Figure:**
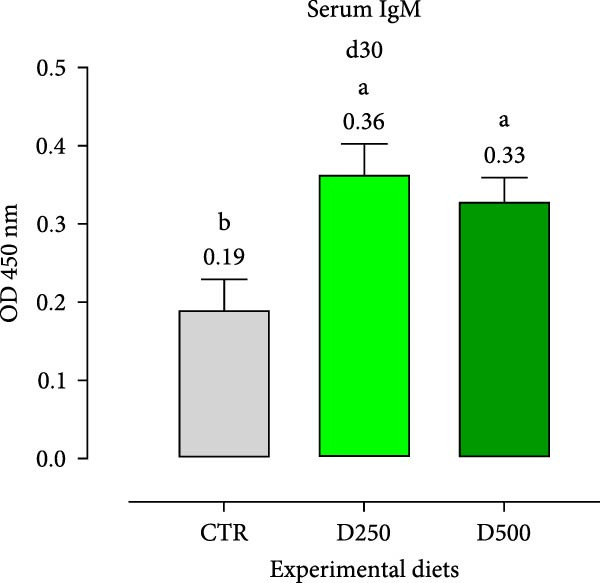
(b)

**Figure:**
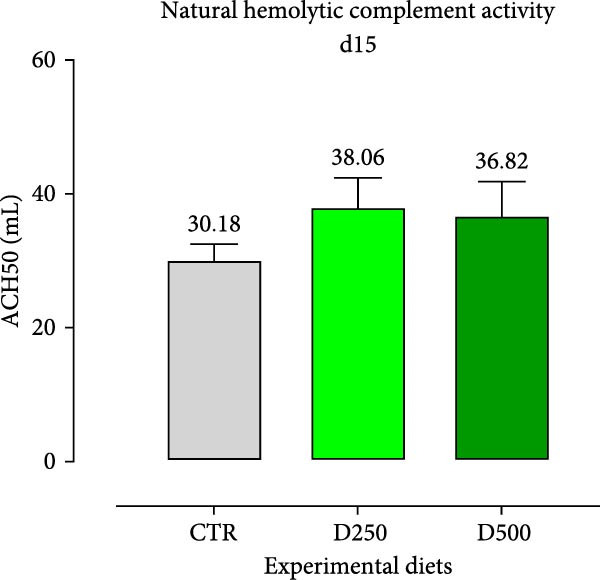
(c)

**Figure:**
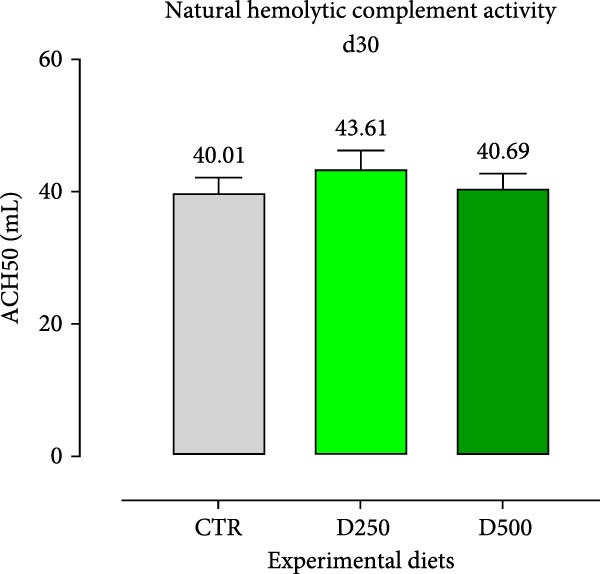
(d)

Regarding IgM, levels in serum from fish fed D250 and D500 for both 15 and 30 days were higher than the values recorded for fish fed the CTR diet (Figure 4).

#### 3.2.3. Cellular Immunity

In relation to the respiratory burst activity of HKL*s*, fish fed D500 for 15 days exhibited a significant enhancement compared to the values observed in HKLs from CTR fish. A comparable pattern was noted after 30 days of the trial, when the activity tended to increase relative to fish maintained on the CTR diet (Figure 5). Furthermore, HKLs isolated from fish fed D500 for 15 days demonstrated a markedly higher phagocytic ability than those from fish on the CTR diet. No significant differences were observed in this parameter for fish at the 30‐day time‐point (Figure 5). More pronounced differences in phagocytosis were evident when assessing phagocytic capacity, which was increased in fish fed D500 after 15 days, with a similar trend (*p* = 0.06) at 30 days, compared to the CTR (Figure 6).

Figure 5Respiratory burst of head‐kidney leukocytes isolated from fish fed for 15 days (a) and 30 days (b) with CTR, D250, or D500 diets. Data in the graph are means (*n* = 5) ± *SEM* represented by vertical bars. Means with different letters indicate statistical significance with *p*  < 0.05 (a, b).

**Figure:**
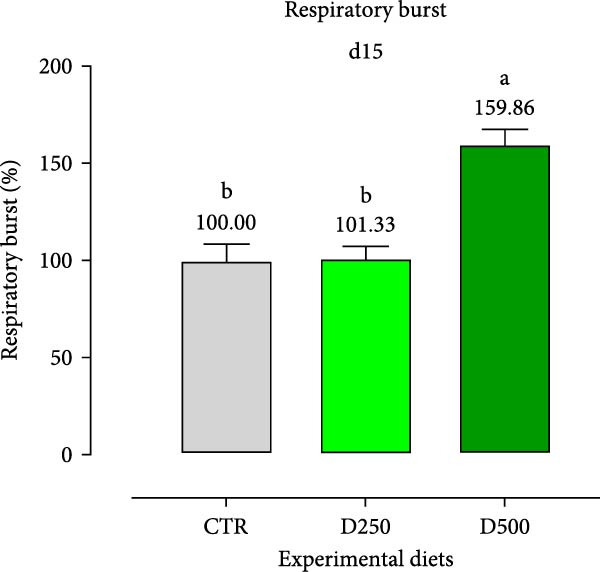
(a)

**Figure:**
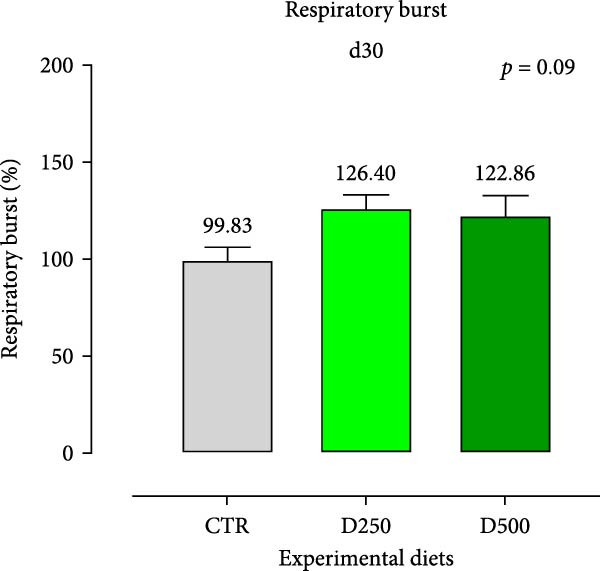
(b)

Figure 6Phagocytic ability and capacity of head‐kidney leukocytes isolated from fish fed for 15 days (a, c) and 30 days (b, d) with CTR, D250, or D500 diets. Data in the graph are means (*n* = 5) ± *SEM* represented by vertical bars. Means with different letters indicate statistical significance with *p*  < 0.05 (a, b).

**Figure:**
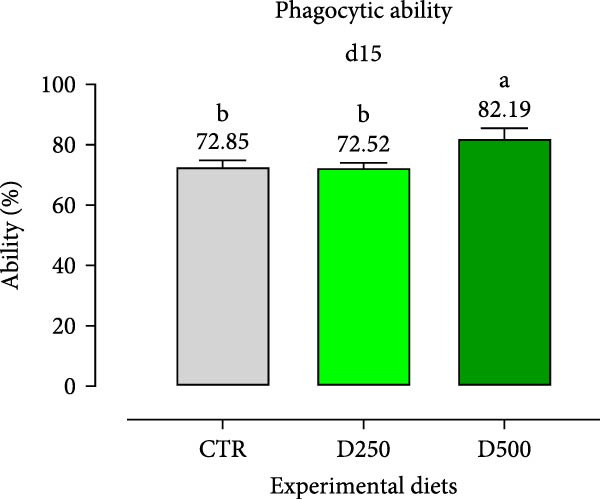
(a)

**Figure:**
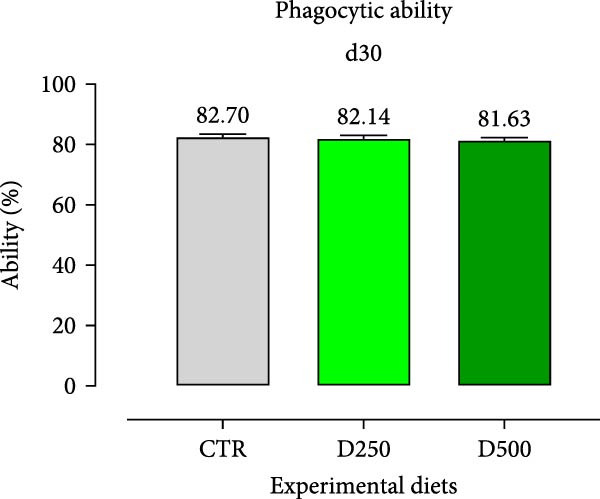
(b)

**Figure:**
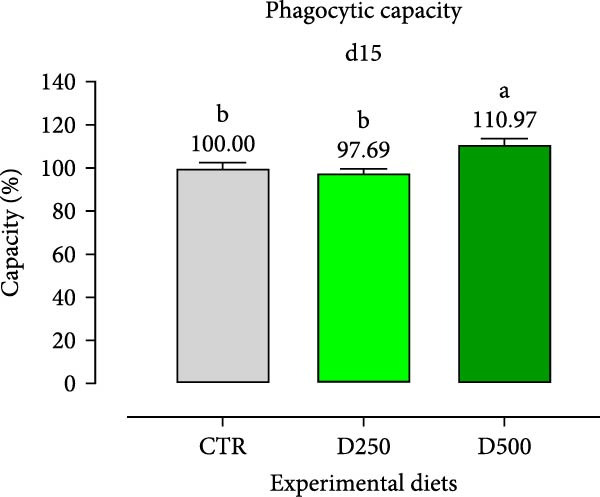
(c)

**Figure:**
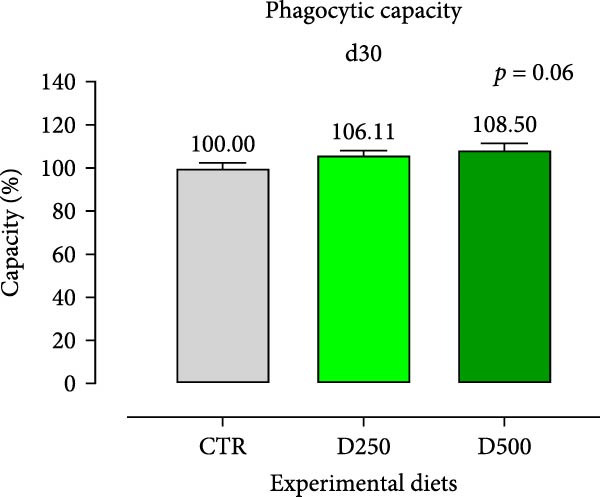
(d)

#### 3.2.4. Gene Expression of Immune Markers

The diets enriched with microencapsulated OA + B blend (D250 and D500) resulted in modulation of mRNA levels of genes involved in oxidative stress and inflammatory responses at the intestinal level. After 15 days, *nrf2* and *sod* were upregulated in fish fed both enriched diets (fold changes of 1.58 and 1.30, respectively), with effects persisting through 30 days. At day 30, both D250 and D500 maintained upregulation of *nrf2* (fold changes of 1.69 and 1.63, respectively) and *sod* (fold changes of 2.64 and 2.31, respectively). Additionally, *gr* showed a significant increase (fold changes 1.84 and 1.90) after 30 days (Figure 7a,b). Regarding pro‐ and anti‐inflammatory ILs expression, fish fed enriched diets D250 and D500 showed modulation already after 15 days. The pro‐inflammatory cytokines *il-7* and *il-8* decreased with both enriched diets. In particular, D250 reduced *il-7* and *il-8* (fold changes of 0.65 and 0.50, respectively), while D500 showed similar effects (fold changes of 0.44 and 0.45, respectively). Conversely, the anti‐inflammatory cytokine *il-10* increased with D500 (fold change of 1.51). These effects were maintained up to day 30 (Figure 7a,b).

Figure 7mRNA levels of antioxidant and inflammatory markers in intestinal samples from fish fed for 15 days (a) and 30 days (b) with CTR, D250, or D500 diets. *nrf2* = nuclear factor erythroid 2; *sod* = superoxide dismutase; *gr* = glutathione reductase; *il* = interleukin. Data in the graph are means (*n* = 5) ± *SEM* represented by vertical bars. Means with different letters indicate statistical significance with *p*  < 0.05 (a, b).

**Figure:**
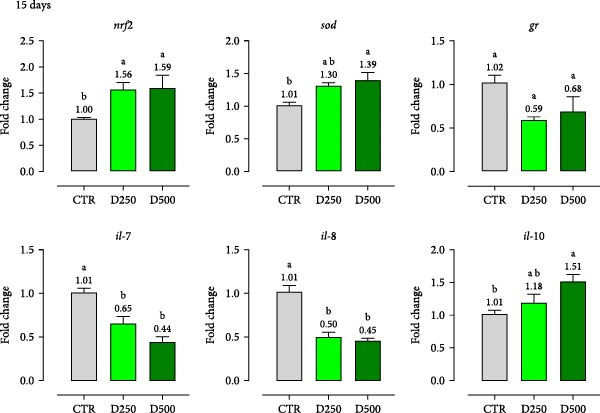
(a)

**Figure:**
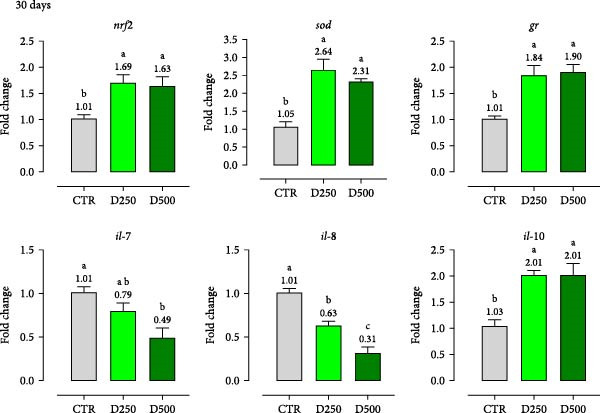
(b)

According to the results from the in vitro test and in vivo phagocytosis, respiratory burst, and gene expression analyses, the blend modulated antioxidant and immunomodulatory defenses when included in fish diets.

## 4. Discussion

In view of the critical role of the aquaculture industry in sustainable food production, it is essential to ensure natural products are able to promote healthier and safer high‐quality seafood [38]. These considerations have hastened the search for dietary supplements or additives able to maintain performance and survival rates under challenging aquaculture conditions [39]. In this context, dietary supplementation with bioactive compounds is generally considered an effective way to sustainably manage fish under stressful farming conditions and environmental fluctuations.

Botanicals and OA have been used for several decades in the terrestrial animal feed industry as antimicrobials [13], antioxidants, and growth and digestion promoters [22, 40]. Following these positive experiences in terrestrial animal feeding, diverse OA and their associated salts have been the focus of research for use in aquatic animal feeds [41, 42]. Also, the inclusion of botanicals not only contributes to fish welfare but also aligns with consumer demand for sustainable and antibiotic‐reduced aquaculture [38, 43]. However, despite the importance of gilthead seabream and the untapped potential of these molecules, the literature on this topic remains limited. For this reason, the main goal of this study was to develop both in vitro and in vivo trials to better understand the potential of a combination of OA + B on cellular and humoral immunity, using gilthead seabream as a marine farmed fish model.

In the present study, the direct impact of the OA + B mixture was first assessed on HKLs. As expected, our results showed that OA + B did not exert any negative effect on cell viability, even at the highest concentration tested, consistently with previous data on Caco‐2 cells [21]. Phagocytosis is an essential initial immune response that involves the ultimate entry of pathogens into phagosomes/lysosomes, leading to the generation of ROS [44]. In this study, HKL phagocytic ability and capacity were positively modulated by the treatment with OA + B. This suggests that the increased phagocytic activity observed was not only related to higher activation of leukocytes, but also to an increased number of phagocytes present in the HKLs. The cellular consumption of molecular oxygen is correlated with the activation of professional phagocytes, which are potent microbial killers in the innate immune system [45]. The improved activation of HKLs observed in the in vitro assay related with the treatment with OA + B was supported by the respiratory burst results, which demonstrated a significant increment in ROS production. Collectively, these findings suggest that all these factors may contribute to a stronger and faster response against pathogen infection, thereby supporting the fish immune system.

Further evidence of OA + B’s ability to modulate the fish immune response is given by the results of gene expression analysis of treated HKLs. For both the time‐points, antioxidant‐related genes were upregulated starting at 100 ppm of inclusion. Moreover, the present study found that OA + B resulted in a modulation outcome of both pro‐inflammatory and anti‐inflammatory cytokines. In light of prior research on how plant extracts affect fish’s oxidative status and immune genes [46, 47], the botanical component of the blend can be credited for the immune‐boosting effect observed in this study. The available results from in vitro studies carried out with fish leukocytes treated with plant extracts [31, 48, 49] also demonstrated modulation of the respiratory burst of leukocytes, including in gilthead seabream. Those studies showed that results varied depending on the plant, extract type, incubation time, and many other factors.

It is well known, particularly in fish species, that results dealing with the effects of immunomodulators or other active substances based on in vitro experiments are important for understanding their direct effects on immune cells in order to exclude possible negative impacts. However, when comparing the effects of the same substances within whole organisms, controversial results can be found. In the present study, the second trial consisted of the incorporation of the microencapsulated blend into the diet of gilthead seabream, in order to determine the effects on immune status and growth‐promoting properties. Regarding immune status and cellular immunity, leukocytes isolated from fish fed D500 for 15 days showed significantly higher phagocytic ability and capacity than those isolated from fish fed the CTR diet. Perhaps, present results could be due to an increase in leukocyte numbers in fish fed supplemented diets. Additionally, the production of ROS measured by the respiratory burst was significantly increased with D500, demonstrating consistency with the data obtained from in vitro tests and supporting the effective role of OA + B in modulating the immune system. After 30 days of trial, the cellular immunity markers of fish fed D500 experienced a similar increase, but only as a trend, suggesting greater efficacy as an initial positive imprinting of the additive. Similarly, studies reported an enhancement of extracellular respiratory burst activity in rainbow trout (*Oncorhynchus mykiss*) fed with ginger [50] and common carp (*Cyprinus carpio*) fed with eucalyptus [51].

Subsequently, we examined specific bioindicators, such as complement activity and IgM, to assess humoral immunity. From a general standpoint, fish fed supplemented diets had higher levels of natural hemolytic complement activity and IgM in serum, although statistically significant increases were only observed in IgM levels for both treated diets (D250 and D500) and at all the time‐points, compared to the values found in fish fed the CTR diet. These results agree with those obtained from [52] in Nile tilapia (*Oreochromis niloticus*) after supplementation with acidifiers (formic and propionic acid salt mixture), and with those obtained by Nhu et al. [53] with plant extracts in striped catfish (*Pangasianodon hypophthalmus*). In the humoral immunity data, we observed that the OA + B blend not only stimulated a robust immune response but also systematically trained the body, enhancing its readiness to effectively combat infections.

To support the speculation of a systemic response, rather than one confined to leukocytes alone, the intestinal immune response was evaluated by studying the expression of several antioxidant and inflammatory markers. NRF2, SOD, and GR are key regulators of the oxidative stress response and protection against ROS. In the present study, fish fed D250 and D500 showed modulation of mRNA levels of *nrf2*, *sod*, and *gr* after 15 and 30 days, suggesting beneficial modulation of the antioxidant system. Again, this result can be explained by the botanical ingredients of the blend, which are widely known as natural antioxidants [54]. The beneficial role of plant extracts as natural antioxidants was reported in Senegalese sole (*Solea senegalensis*) [55], with modulation of gene expression using green tea and grape extract [55]. Oxidative stress and inflammation are pathological processes that are closely linked and can easily be triggered by each other. To CTR an undesired inflammatory situation, it is essential to act on both of these factors. The OA + B mixture not only positively modulates oxidative‐related genes, but also produces parallel modulation of mRNA levels of pro‐inflammatory (*il-7* and *il-8*) and anti‐inflammatory (*il-10*) markers at the intestinal level, confirming in vitro data. Similarly, previous results in European sea bass (*Dicentrarchus labrax*) also reported positive modulation in anti‐inflammatory functions [25].

Finally, as expected based on the standards of this aquatic species and the parameters of the experimental environment, in the present study, the weight of the fish increased over time during the experiment, more than doubling in size, with differences between treatments. Growth performance was positively influenced by OA + B at the highest dose (D500). Regarding SGR performance, a remarkable result was that fish fed both supplemented diets had higher SGR than fish fed the CTR diet at 30 and 60 days into the trial: diet D500 reached statistical significance at both time‐points, while diet D250 had more fluctuating results with a stronger effect at 30 days. These growth patterns highlight that the OA + B blend requires a minimum supplementation period of 2–4 weeks before effects on growth dynamics can be measured. With respect to the role of OA + B in promoting growth, this can be, on one hand, attributed to the blend’s ameliorating and anti‐inflammatory direct effect on the intestinal epithelial cells [21]; on the other hand, although the scientific literature for gilthead seabream remains very limited, to beneficial modulation of the gastrointestinal microflora [26], particularly regarding the phytogenic blend under investigation, although OA have been deeply studied [56]. Citric acid has demonstrated consistent benefits across species; for example, in rainbow trout and yellow catfish (*Pelteobagrus fulvidraco*), its inclusion enhanced phosphorus absorption, mineral bioavailability, and growth performance [57, 58]. Concerning thymol effect on seabream, Firmino et al. [59] evaluated the impact of a diet supplemented with garlic essential oil, carvacrol, and thymol on seabream juveniles without reporting significant improvements in growth performance, while a different study using a thymol and carvacrol blend showed anti‐inflammatory and anti‐proliferative responses and modulation in feed conversion ratio in seabream juveniles [51]. On the other hand, our previous research demonstrated that dietary inclusion of a blend containing OA + B improved growth performance and nutrient utilization in rainbow trout, but similar effects have not been consistently observed in European seabass, where immune modulation and microbiota shift with positive repercussions on overall health were observed [25, 26]. These findings suggest that botanical molecules are consistently able to support immune function and overall health, but may additionally promote growth when in combination with OA. This concept is further supported by data from terrestrial livestock species, where microencapsulated additives based on sorbic acid, citric acid, thymol, and vanillin enhanced in vitro intestinal integrity and barrier function and in vivo intestinal nutrient digestion and transporter expression, and function [21, 24].

## 5. Conclusions

In conclusion, the present results demonstrate that a microencapsulated blend of sorbic acid, citric acid, thymol, and vanillin (OA + B) exerts consistent immunomodulatory and growth‐promoting effects in gilthead seabream. In vitro, the blend preserved leukocyte viability while enhancing respiratory burst activity, phagocytic capacity, and antioxidant gene expression, indicating the blend’s potential to boost immune function. When administered through dietary inclusion to gilthead seabream juveniles in vivo, the blend induced increased phagocytic activity, ROS production, and serum IgM levels after 15 days, with trends persisting at 30 days, indicating an early and sustained stimulation of cellular and humoral immunity. The upregulation of antioxidant genes (*nrf2*, *sod*, *gr*) and the balanced modulation of pro‐ and anti‐inflammatory cytokines (*il-7*, *il-8*, *il-10*) further support the role of OA + B in managing oxidative stress and immune activation at the intestinal level.

Performance metrics improved in parallel with the immune modulation, with notable enhancements observed at 500 ppm of inclusion after 30 and 60 days of feeding, while 250 ppm induced intermediate and more variable responses. These findings highlight a dose‐ and time‐dependent efficacy of the blend, with a minimal supplementation period of 2–4 weeks required to observe consistent growth improvements. Collectively, these results demonstrate that dietary OA + B supplementation modulates immune homeostasis and promotes growth in gilthead seabream juveniles under nonchallenging conditions. Further investigation into immune parameters is necessary to elucidate the mechanisms linking immunomodulation, intestinal health, and growth performances. Challenge studies are recommended to comprehensively characterize the blend’s efficacy in vivo under challenging or stressful conditions.

## Conflicts of Interest

Andrea Piva reports a relationship with Vetagro Spa that includes board membership. Ester Grilli reports a relationship with Vetagro Inc. that includes board membership. Ester Grilli reports a relationship with the University of Bologna that includes employment. Fabrizio Caruso and Andrea Toschi report a relationship with Vetagro S.p.A. that includes employment.

## Author Contributions

Prof. Andrea Piva, Dr. Ester Grilli, and Prof. María Ángeles Esteban conceptualized and supervised the research. Prof. María Ángeles Esteban, Dr. José María García‐Beltrán, and Prof. Alberto Cuesta performed the experiments. Costanza Bonnici, Dr. Andrea Toschi, and Fabrizio Caruso carried out feed validation and performed the statistical analysis. Fabrizio Caruso and Dr. Andrea Toschi wrote the original draft of the manuscript. Prof. María Ángeles Esteban reviewed the manuscript. All authors provided critical feedback and helped shape the research, analysis, and the final manuscript.

## Funding

This research was financially supported by Emilia Romagna Region, Italy, “Bando per progetti collaborativi di ricerca e sviluppo delle imprese (DGR Number 773/2015), Asse 1 ‐ Azione 1.1.1. e Azione 1.1.4 del POR‐FESR Emilia Romagna 2014–2020,” Project Number PG/2015/794448, CUP code E88C1. This research forms part of the ThinkInAzul Programme and was supported by MCIN with funding from European Union Next Generation EU (PRTR‐C17.I01) and by Comunidad Autónoma de la Región de Murcia – Fundación Séneca.

## Supporting Information

Additional supporting information can be found online in the Supporting Information section.

## Supporting information


**Supporting Information 1** The following are available online. Table S1. Analytical composition of commercial diet VITA 2 by Veronesi, A.I.A. Spa. Table S2. Primers used for qPCR analysis on gilthead seabream samples. Figure S1. Viability of HK leukocytes treated for 30 min, 2 h, and 4 h with incremental doses of the blend of OA+B.

**Supporting Information 2** Figure S2. Graphical abstract.

## Data Availability

Data are available upon request to the corresponding author.

## References

[1] FAO , The State of World Fisheries and Aquaculture, 2024.

[2] Basurco B. , Lovatelli A. , and García B. , Current Status of Sparidae Aquaculture, Sparidae, 2011, 1 edition, Wiley, 1–50.

[3] Sánchez-Muros M. J. , Sánchez B. , Barroso F. G. , Toniolo M. , Trenzado C. E. , and Rus A. S. , Effects of Rearing Conditions on Behavioural Responses, Social Kinetics and Physiological Parameters in Gilthead Sea Bream Sparus Aurata, Applied Animal Behaviour Science. (2017) 197, 120–128, 10.1016/j.applanim.2017.08.004, 2-s2.0-85028342320.

[4] FAO , The State of Mediterranean and Black Sea Fisheries, 2023.

[5] Ashley P. J. , Fish Welfare: Current Issues in Aquaculture, Applied Animal Behaviour Science. (2007) 104, no. 3-4, 199–235, 10.1016/j.applanim.2006.09.001, 2-s2.0-34047120584.

[6] Vendramin N. , Zrncic S. , and Padrós F. , et al.Fish Health in Mediterranean Aquaculture, Past Mistakes and Future Challenges, Bulletin of the European Association of Fish Pathologists. (2016) 36, no. 1, 38–45.

[7] Muniesa A. , Basurco B. , and Aguilera C. , Mapping the Knowledge of the Main Diseases Affecting Sea Bass and Sea Bream in Mediterranean, Transboundary and Emerging Diseases. (2020) 67, no. 3, 1089–1100, 10.1111/tbed.13482. 31960605

[8] Oliva-Teles A. , Nutrition and Health of Aquaculture Fish, Journal of Fish Diseases. (2012) 35, no. 2, 83–108, 10.1111/j.1365-2761.2011.01333.x, 2-s2.0-84855792431. 22233511

[9] Pohlenz C. and Gatlin D. M. , Interrelationships Between Fish Nutrition and Health, Aquaculture. (2014) 431, 111–117, 10.1016/j.aquaculture.2014.02.008, 2-s2.0-84905040281.

[10] Encarnação P. , Functional Feed Additives in Aquaculture Feeds, Aquafeed Formulation, 2016, Elsevier, 217–237.

[11] Vijayaram S. , Sun Y.-Z. , Zuorro A. , Ghafarifarsani H. , Van Doan H. , and Hoseinifar S. H. , Bioactive Immunostimulants as Health-Promoting Feed Additives in Aquaculture: A Review, Fish & Shellfish Immunology. (2022) 130, 294–308, 10.1016/j.fsi.2022.09.011. 36100067

[12] Sutili F. J. , Gatlin D. M.III, Heinzmann B. M. , and Baldisserotto B. , Plant Essential Oils as Fish Diet Additives: Benefits on Fish Health and Stability in Feed, Reviews in Aquaculture. (2018) 10, no. 3, 716–726, 10.1111/raq.12197, 2-s2.0-85017402197.

[13] Rossi B. , Toschi A. , Piva A. , and Grilli E. , Single Components of Botanicals and Nature-Identical Compounds as a Non-Antibiotic Strategy to Ameliorate Health Status and Improve Performance in Poultry and Pigs, Nutrition Research Reviews. (2020) 33, no. 2, 218–234, 10.1017/S0954422420000013. 32100670

[14] García Beltrán J. M. and Esteban M. Á. , Nature-Identical Compounds as Feed Additives in Aquaculture, Fish & Shellfish Immunology. (2022) 123, 409–416, 10.1016/j.fsi.2022.03.010. 35331881

[15] Zhang R. , Kang X. , and Liu L. , Gut Microbiota Modulation by Plant Polyphenols in Koi Carp (*Cyprinus carpio* L.), Frontiers in Microbiology. (2022) 13, 10.3389/fmicb.2022.977292. PMC9597254 36312947

[16] Cherukumudi K. , Augustian H. T. , and Kausar N. , et al.Exploring Botanical Innovations in Fish Aquaculture: A Review of Biological Impacts and Industry Prospects: Exploring Botanical Innovations in Fish Aquaculture, Fishery Technology. (2024) 61, no. 4, 10.56093/ft.v61i4.146588.

[17] Benedito-Palos L. , Ballester-Lozano G. F. , and Simó P. , Lasting Effects of Butyrate and Low FM/FO Diets on Growth Performance, Blood Haematology/Biochemistry and Molecular Growth-Related Markers in Gilthead Sea Bream (*Sparus aurata*), Aquaculture. (2016) 454, 8–18, 10.1016/j.aquaculture.2015.12.008, 2-s2.0-84949814504.

[18] Piazzon M. C. , Calduch-Giner J. A. , and Fouz B. , et al.Under Control: How a Dietary Additive can Restore the Gut Microbiome and Proteomic Profile, and Improve Disease Resilience in a Marine Teleostean Fish Fed Vegetable Diets, Microbiome. (2017) 5, no. 1, 10.1186/s40168-017-0390-3, 2-s2.0-85053916552. PMC5745981 29282153

[19] Simó-Mirabet P. , Perera E. , Calduch-Giner J. A. , Afonso J. M. , and Pérez-Sánchez J. , Co-Expression Analysis of Sirtuins and Related Metabolic Biomarkers in Juveniles of Gilthead Sea Bream (*Sparus aurata*) With Differences in Growth Performance, Frontiers in Physiology. (2018) 9, 10.3389/fphys.2018.00608, 2-s2.0-85048031851. PMC5996159 29922168

[20] Grilli E. , Tugnoli B. , and Passey J. L. , et al.Impact of Dietary Organic Acids and Botanicals on Intestinal Integrity and Inflammation in Weaned Pigs, BMC Veterinary Research. (2015) 11, no. 1, 10.1186/s12917-015-0410-0, 2-s2.0-85019259780, 96. 25889654 PMC4483210

[21] Toschi A. , Rossi B. , Tugnoli B. , Piva A. , and Grilli E. , Nature-Identical Compounds and Organic Acids Ameliorate and Prevent the Damages Induced by an Inflammatory Challenge in Caco-2 Cell Culture, Molecules. (2020) 25, no. 18, 10.3390/molecules25184296, 4296. 32961674 PMC7570934

[22] Tugnoli B. , Giovagnoni G. , Piva A. , and Grilli E. , From Acidifiers to Intestinal Health Enhancers: How Organic Acids can Improve Growth Efficiency of Pigs, Animals. (2020) 10, no. 1, 10.3390/ani10010134, 134. 31947627 PMC7022919

[23] Bialkowski S. , Toschi A. , and Yu L.-E. , Effects of Microencapsulated Blend of Organic Acids and Botanicals on Growth Performance, Intestinal Barrier Function, Inflammatory Cytokines, and Endocannabinoid System Gene Expression in Broiler Chickens, Poultry Science. (2023) 102, no. 3, 10.1016/j.psj.2022.102460, 102460. PMC10014334 36680863

[24] Toschi A. , Yu L.-E. , Bialkowski S. , Schlitzkus L. , Grilli E. , and Li Y. , Dietary Supplementation of Microencapsulated Botanicals and Organic Acids Enhances the Expression and Function of Intestine Epithelial Digestive Enzymes and Nutrient Transporters in Broiler Chickens, Poultry Science. (2024) 103, no. 11, 10.1016/j.psj.2024.104237, 104237. PMC11402617 39217663

[25] Busti S. , Rossi B. , and Volpe E. , et al.Effects of Dietary Organic Acids and Nature Identical Compounds on Growth, Immune Parameters and Gut Microbiota of European Sea Bass, Scientific Reports. (2020) 10, no. 1, 10.1038/s41598-020-78441-9. PMC7721706 33288837

[26] Pelusio N. F. , Rossi B. , and Parma L. , Effects of Increasing Dietary Level of Organic Acids and Nature-Identical Compounds on Growth, Intestinal Cytokine Gene Expression and Gut Microbiota of Rainbow Trout (*Oncorhynchus mykiss*) Reared at Normal and High Temperature, Fish & Shellfish Immunology. (2020) 107, 324–335, 10.1016/j.fsi.2020.10.021. 33096247

[27] He W. , Rahimnejad S. , Wang L. , Song K. , Lu K. , and Zhang C. , Effects of Organic Acids and Essential Oils Blend on Growth, Gut Microbiota, Immune Response and Disease Resistance of Pacific White Shrimp (Litopenaeus Vannamei) Against Vibrio Parahaemolyticus, Fish & Shellfish Immunology. (2017) 70, 164–173, 10.1016/j.fsi.2017.09.007, 2-s2.0-85028925860. 28882791

[28] National Research Council , Nutrient Requirements of Fish and Shrimp, 2011, National Academies Press, Washington, D.C..

[29] Leary S. , AVMA Guidelines for the Euthanasia of Animals, 2020, 2020 edition.

[30] Esteban M. A. , Mulero V. , Muñoz J. , and Meseguer J. , Methodological Aspects of Assessing Phagocytosis of *Vibrio anguillarum* by Leucocytes of Gilthead Seabream (*Sparus aurata L*.) by Flow Cytometry and Electron Microscopy, Cell and Tissue Research. (1998) 293, no. 1, 133–141, 10.1007/s004410051105, 2-s2.0-0031851921. 9634605

[31] Fazio A. , Cerezuela R. , Panuccio M. R. , Cuesta A. , and Esteban M. , In Vitro Effects of Italian, *Lavandula multifida*, L. Leaf Extracts on Gilthead Seabream (*Sparus aurata*) Leucocytes and SAF-1 Cells, Fish & Shellfish Immunology. (2017) 66, 334–344, 10.1016/j.fsi.2017.05.033, 2-s2.0-85019605029. 28522420

[32] Özdemir A. , Şanlı S. , Sardoğan B. , and Sardoğan S. , Determination of Sorbic Acid in Cheese Samples by Rapid HPLC-DAD Method, International Journal of Analytical Chemistry. (2020) 2020, no. 1, 10.1155/2020/6049028, 6049028. 32411248 PMC7204100

[33] Silva-Carrillo Y. , Hernández C. , Hardy R. W. , González-Rodríguez B. , and Castillo-Vargasmachuca S. , The Effect of Substituting Fish Meal With Soybean Meal on Growth, Feed Efficiency, Body Composition and Blood Chemistry in Juvenile Spotted Rose Snapper Lutjanus Guttatus (Steindachner, 1869), Aquaculture. (2012) 364, 180–185, 10.1016/j.aquaculture.2012.08.007, 2-s2.0-84865648609.

[34] Rodríguez A. , Esteban M. A. , and Meseguer J. , A Mannose-Receptor is Possibly Involved in the Phagocytosis of *Saccharomyces cerevisiae* by Seabream (*Sparus aurata L*.) Leucocytes, Fish & Shellfish Immunology. (2003) 14, no. 5, 375–388, 10.1006/fsim.2002.0446, 2-s2.0-0242418168. 12711272

[35] Cuesta A. , Meseguer J. , and Esteban M. A. , Total Serum Immunoglobulin M Levels are Affected by Immunomodulators in Seabream (*Sparus aurata* L.), Veterinary Immunology and Immunopathology. (2004) 101, no. 3-4, 203–210, 10.1016/j.vetimm.2004.04.021, 2-s2.0-4444256158. 15350750

[36] Bayne C. J. and Levy S. , Modulation of the Oxidative Burst in Trout Myeloid Cells by Adrenocorticotropic Hormone and Catecholamines: Mechanisms of Action, Journal of Leukocyte Biology. (1991) 50, no. 6, 554–560, 10.1002/jlb.50.6.554, 2-s2.0-0026045649. 1658172

[37] Livak K. J. and Schmittgen T. D. , Analysis of Relative Gene Expression Data Using Real-Time Quantitative PCR and the 2–ΔΔCT Method, Methods. (2001) 25, no. 4, 402–408, 10.1006/meth.2001.1262, 2-s2.0-0035710746. 11846609

[38] Bondad-Reantaso M. G. , MacKinnon B. , and Karunasagar I. , Review of Alternatives to Antibiotic Use in Aquaculture, Reviews in Aquaculture. (2023) 15, no. 4, 1421–1451, 10.1111/raq.12786.

[39] Bai S. C. , Katya K. , and Yun H. , Additives in Aquafeed: An Overview, 7, *Feed and Feeding Practices in Aquaculture*, 2015, Woodhead Publishing, 171–202.

[40] Kluge H. , Broz J. , and Eder K. , Effect of Benzoic Acid on Growth Performance, Nutrient Digestibility, Nitrogen Balance, Gastrointestinal Microflora and Parameters of Microbial Metabolism in Piglets, Journal of Animal Physiology and Animal Nutrition. (2006) 90, no. 7-8, 316–324, 10.1111/j.1439-0396.2005.00604.x, 2-s2.0-33748354018. 16867077

[41] Ng W.-K. and Koh C.-B. , The Utilization and Mode of Action of Organic Acids in the Feeds of Cultured Aquatic Animals, Reviews in Aquaculture. (2017) 9, no. 4, 342–368, 10.1111/raq.12141, 2-s2.0-84959267905.

[42] Sukor S. A. , Taher S. , Ehteshamei F. , Arshad A. , Ng W.-K. , and Romano N. , Effects of Different Dietary Organic Acids on the Survival, Growth, and Hepatopancreatic Histopathology of the Blue Swimmer Crab *Portunus pelagicus* , Journal of Shellfish Research. (2016) 35, no. 2, 555–561, 10.2983/035.035.0228, 2-s2.0-84987677734.

[43] Hernández Serrano P. , Responsible Use of Antibiotics in Aquaculture, 2005, FAO Fisheries Technical Paper, Rome, FAO.

[44] Gartlan K. H. , Krashias G. , and Wegmann F. , Sterile Inflammation Induced by Carbopol Elicits Robust Adaptive Immune Responses in the Absence of Pathogen-Associated Molecular Patterns, Vaccine. (2016) 34, no. 19, 2188–2196, 10.1016/j.vaccine.2016.03.025, 2-s2.0-84963616088. 27005810 PMC4850248

[45] Dahlgren C. , Karlsson A. , and Bylund J. , Measurement of Respiratory Burst Products Generated by Professional Phagocytes, Neutrophil Methods and Protocols (a c. diQuinn, 2007, Humana Press, Totowa, NJ, 349–363. 10.1007/978-1-59745-467-4_23 18453123

[46] Abdel-Latif H. M. R. , Abdel-Tawwab M. , Khafaga A. F. , and Dawood M. A. O. , Dietary Origanum Essential Oil Improved Antioxidative Status, Immune-Related Genes, and Resistance of Common Carp (*Cyprinus carpio* L.) to Aeromonas Hydrophila Infection, Fish & Shellfish Immunology. (2020) 104, 1–7, 10.1016/j.fsi.2020.05.056. 32474085

[47] Brum A. , Pereira S. A. , and Owatari M. S. , et al.Effect of Dietary Essential Oils of Clove Basil and Ginger on Nile Tilapia (*Oreochromis niloticus*) Following Challenge With *Streptococcus agalactiae* , Aquaculture. (2017) 468, 235–243, 10.1016/j.aquaculture.2016.10.020, 2-s2.0-84992028905.

[48] Henry M. A. , Nikolopoulou D. , and Alexis M. N. , In Vitro Effect of Peas, Pisum Pisum, and Chickpeas, Cicer Arietinum, on the Immune System of Gilthead Seabream, Sparus Aurata, Vitro Cellular & Developmental Biology - Animal. (2012) 48, no. 7, 10.1007/s11626-012-9528-6, 2-s2.0-84867008629, 412. 22752638

[49] Bulfon C. , Galeotti M. , and Volpatti D. , Medicinal Plant Extracts Modulate Respiratory Burst and Proliferation Activity of Rainbow Trout (*Oncorhynchus mykiss*) Leukocytes, Fish Physiology and Biochemistry. (2018) 44, no. 1, 109–117, 10.1007/s10695-017-0417-5, 2-s2.0-85028761880. 28861641

[50] Dügenci S. K. , Arda N. , and Candan A. , Some Medicinal Plants as Immunostimulant for Fish, Journal of Ethnopharmacology. (2003) 88, no. 1, 99–106, 10.1016/S0378-8741(03)00182-X, 2-s2.0-0041666419. 12902058

[51] Dawood M. A. O. , El Basuini M. F. , and Yilmaz S. , et al.Exploring the Roles of Dietary Herbal Essential Oils in Aquaculture: A Review, Animals. (2022) 12, no. 7, 10.3390/ani12070823. PMC8996993 35405814

[52] Reda R. M. , Mahmoud R. , Selim K. M. , and El-Araby I. E. , Effects of Dietary Acidifiers on Growth, Hematology, Immune Response and Disease Resistance of Nile Tilapia, *Oreochromis niloticus* , Fish & Shellfish Immunology. (2016) 50, 255–262, 10.1016/j.fsi.2016.01.040, 2-s2.0-84957871189. 26860238

[53] Nhu T. Q. , Bich Hang B. T. , and Bach L. T. , Plant Extract-Based Diets Differently Modulate Immune Responses and Resistance to Bacterial Infection in Striped Catfish (*Pangasianodon hypophthalmus*), Fish & Shellfish Immunology. (2019) 92, 913–924, 10.1016/j.fsi.2019.07.025, 2-s2.0-85068907794. 31306761

[54] Toschi A. , Piva A. , and Grilli E. , Phenol-Rich Botanicals Modulate Oxidative Stress and Epithelial Integrity in Intestinal Epithelial Cells, Animals. (2022) 12, no. 17, 10.3390/ani12172188, 2188. 36077907 PMC9454507

[55] Xavier M. J. , Conceição L. E. , and Valente L. M. , et al.Dietary Natural Plant Extracts can Promote Growth and Modulate Oxidative Status of Senegalese Sole Postlarvae Under Standard/Challenge Conditions, Animals. (2021) 11, no. 5, 10.3390/ani11051398, 1398. 34068939 PMC8156806

[56] Fabay R. , Serrano Jr A. , Alejos M. , and Fabay J. , Effects of Dietary Acidification and Acid Source on Fish Growth and Feed Efficiency (Review), World Academy of Sciences Journal. (2022) 4, no. 3, 10.3892/wasj.2022.156.

[57] Zhao T. , Xu J.-J. , and Kotzamanis Y. P. , Effects of Dietary Citric Acid on Growth Performance, Mineral Status, Body and Muscle Composition, Muscle Growth and mTOR Signaling in Yellow Catfish *Pelteobagrus fulvidraco* Fed With Low-Manganese Diets, Aquaculture. (2024) 582, 10.1016/j.aquaculture.2024.740569, 740569.

[58] Hernández A. J. , Satoh S. , and Kiron V. , The Effect of Citric Acid Supplementation on Growth Performance, Phosphorus Absorption and Retention in Rainbow Trout (*Oncorhynchus mykiss*) Fed a Low-Fishmeal Diet, Ciencia e Investigación Agraria, 2013, 10.4067/S0718-16202013000200014, 2-s2.0-84886697264.

[59] Firmino J. P. , Galindo-Villegas J. , Reyes-López F. E. , and Gisbert E. , Phytogenic Bioactive Compounds Shape Fish Mucosal Immunity, Frontiers in Immunology. (2021) 12, 10.3389/fimmu.2021.695973. PMC8252966 34220858

