# Supplementary material for: Microencapsulated Botanicals and Organic Acids Improve Immune Status and Growth in Gilthead Seabream (Sparus aurata L.)
Source: Aquac Nutr. 2026 Jan 7;2026:4213038. doi: 10.1155/anu/4213038 (PMC12779933; doi:10.1155/anu/4213038)
Supplement: Supplementary file 1 — Supporting Information 1 The following are available online. Table S1. Analytical composition of commercial diet VITA 2 by Veronesi, A.I.A. Spa. Table S2. Primers used for qPCR analysis on gilthead seabream samples. Figure S1. Viability of HK leukocytes treated for 30 min, 2 h, and 4 h with incremental doses of the blend of OA+B. [file ANU-2026-4213038-s002.docx]

Table S1. Analytical composition of commercial diet VITA 2 by Veronesi, A.I.A. Spa, Verona, Italy for gilthead seabream. Values provided by the supplier.

| **Diet** | **VITA 2** | |
| --- | --- | --- |
| Target species | Gilthead seabream | |
| Feed size | 2,3 - 2,6 | mm |
| Crude protein | 49,8 | % |
| Crude fats and oils | 21,6 | % |
| Crude fiber | 1,5 | % |
| Crude ash | 7,3 | % |
| Total carbohidrates | 10,5 | % |
| Phosphorous | 1,1 | % |
| Vitamin C | 400 | mg/kg |
| Vitamin E | 300 | mg/kg |
| Digestible energy | 19,45 | mj/kg |
|  |  |  |

Table S2. Primers used for qPCR analysis on *S. aurata* samples.

| **Function** | **Gene** | **Forward primer** | **Reverse Primer** | **GenBank no.** |
| --- | --- | --- | --- | --- |
| Housekeeping | ef1α | TGTCATCAAGGCTGTTGAGC | GCACACTTCTTGTTGCTGGA | AF184170 |
|  | 18S | CGAAAGCATTTGCCAAGAAT | AGTTGGCACCGTTTATGGTC | AM490061 |
| Oxidative stress response | nrf2 | GTTCAGTCGGTGCTTTGACA | CTCTGATGTGCGTCTCTCCA | FP335773 |
|  | sod | CCATGGTAAGAATCATGGCGG | CGTGGATCACCATGGTTCTG | AJ937872 |
|  | cat | TTCCCGTCCTTCATTCACTC | CTCCAGAAGTCCCACACCAT | FG264808 |
|  | gr | CAAAGCGCAGTGTGATTGTGG | CCACTCCGGAGTTTTGCATTTC | AJ937873 |
| Pro-inflammatory | il-7 | GATCTGGAAAACACCGGAGA | TGGACGTGCAGTTCTGTAGC | JX976618 |
|  | il-8 | GCCACTCTGAAGAGGACAGG | TTTGGTTGTCTTTGGTCGAA | AM765841 |
| Anti-inflammatory | il-10 | CTCACATGCAGTCCATCCAG | TGTGATGTCAAACGGTTGCT | FG261948 |

F = forward; R = reverse; ef1α = elongation factor 1-alpha; Nrf2 = nuclear factor erythroid 2; SOD = superoxide dismutase; CAT = catalase; GR = glutathione reductase; IL = interleukin.


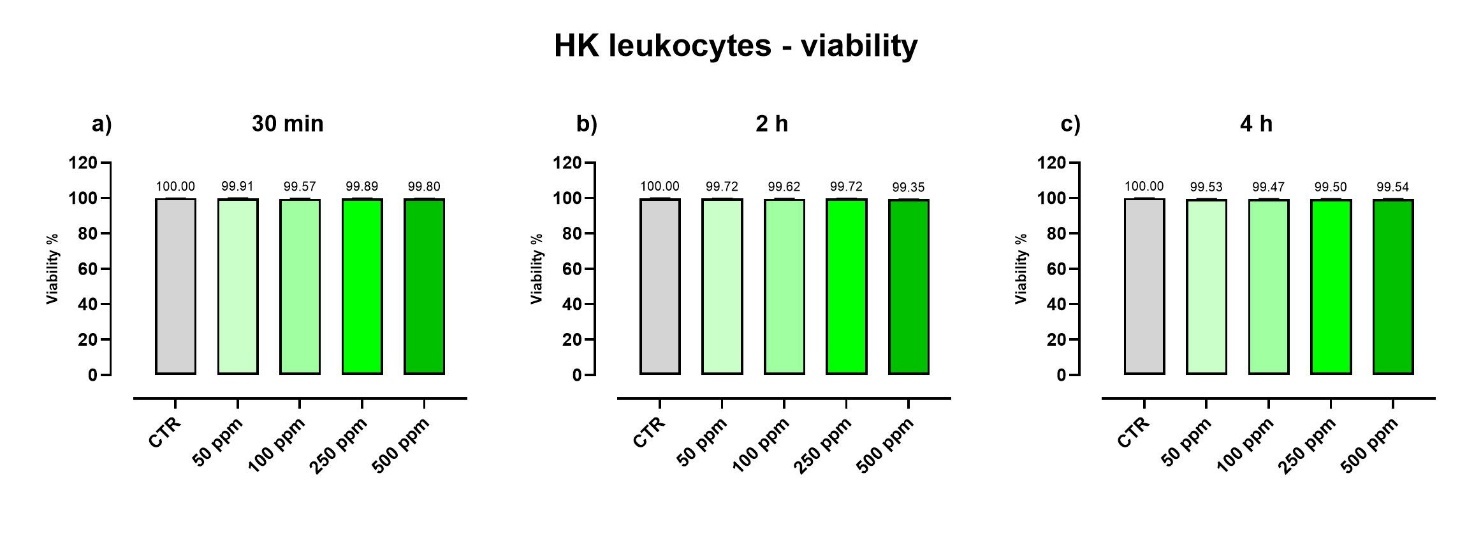


Figure S1. Viability of HK leukocytes treated for 30 min, 2 h, and 4 h with incremental doses of the blend of OA+B. Data in the graph are means (n = 5) ± SEM represented by vertical bars.
