# Supplementary material for: Microencapsulated Botanicals and Organic Acids Improve Immune Status and Growth in Gilthead Seabream (Sparus aurata L.)
Source: Aquac Nutr. 2026 Jan 7;2026:4213038. doi: 10.1155/anu/4213038 (PMC12779933; doi:10.1155/anu/4213038)
Supplement: Supplementary file 2 — Supporting Information 2 Figure S2. Graphical abstract. [file ANU-2026-4213038-s001.pdf]

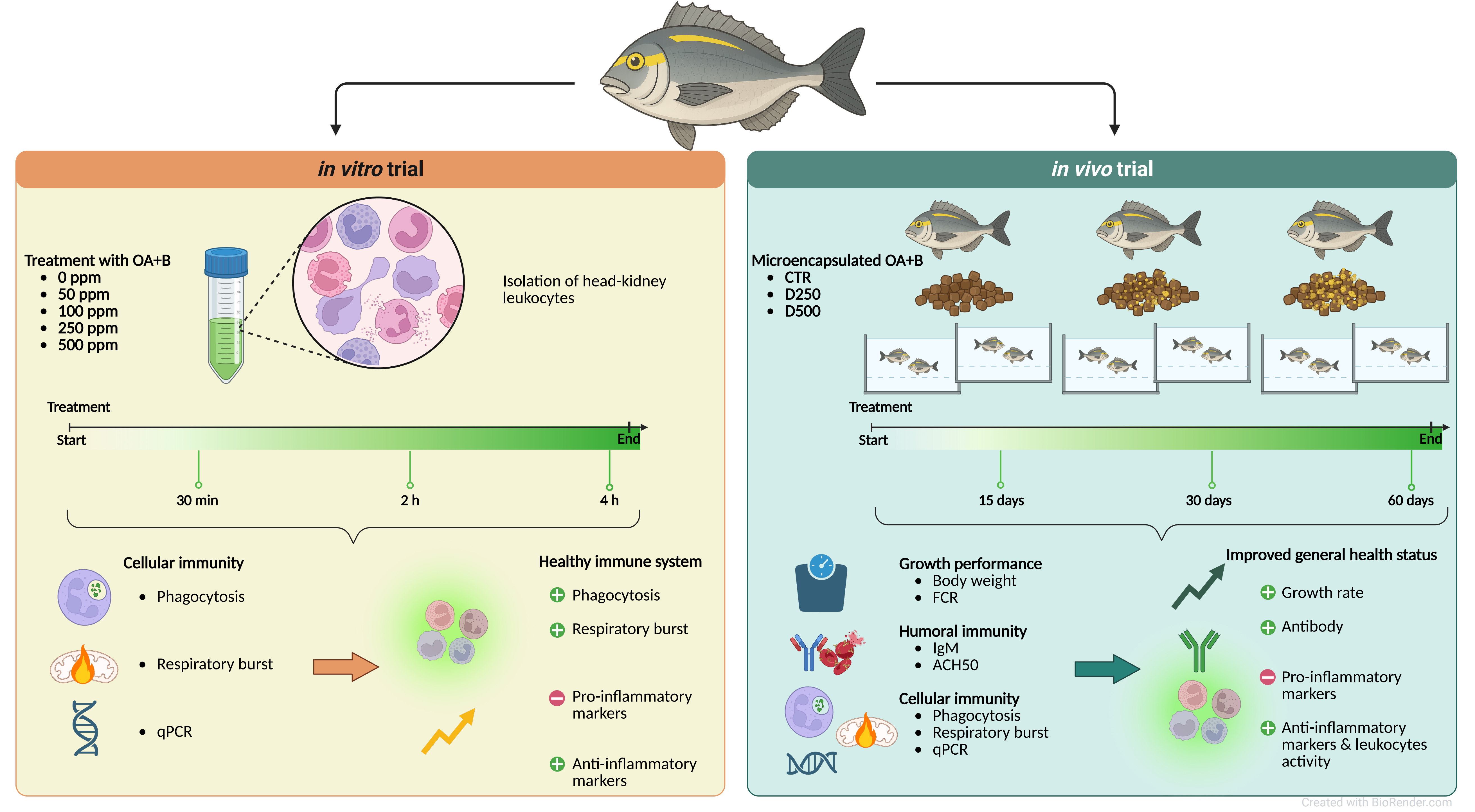

in vitro trial
Treatment with OA+B
• 0 ppm
• 50 ppm
• 100 ppm
• 250 ppm
• 500 ppm
Isolation of head-kidney leukocytes
Treatment
Start
End
30 min
2 h
4 h
Cellular immunity
• Phagocytosis
• Respiratory burst
• qPCR
Healthy immune system
+ Phagocytosis
+ Respiratory burst
− Pro-inflammatory markers
+ Anti-inflammatory markers
in vivo trial
Microencapsulated OA+B
• CTR
• D250
• D500
Treatment
Start
End
15 days
30 days
60 days
Growth performance
• Body weight
• FCR
Humoral immunity
• IgM
• ACH50
Cellular immunity
• Phagocytosis
• Respiratory burst
• qPCR
Improved general health status
+ Growth rate
+ Antibody
− Pro-inflammatory markers
+ Anti-inflammatory markers & leukocytes activity
Created with BioRender.com
